# Retracted: Treadmill Training Increases SIRT-1 and PGC-1*α* Protein Levels and AMPK Phosphorylation in Quadriceps of Middle-Aged Rats in an Intensity-Dependent Manner

**DOI:** 10.1155/2017/8287646

**Published:** 2017-10-16

**Authors:** Mediators of Inflammation

Mediators of Inflammation has retracted the article titled “Treadmill Training Increases SIRT-1 and PGC-1*α* Protein Levels and AMPK Phosphorylation in Quadriceps of Middle-Aged Rats in an Intensity-Dependent Manner” [1] due to unintentional reuse of a previously published image. After a concern was raised to them by a reader, the authors contacted the journal to replace the anti-ACC blot in Figure 2(b), providing the original figure. The journal found that the published blot in Figure 2(b) was similar in other articles [2–9]. Due to this, the journal reassessed the other figures and found that, for the *β*-actin bands in Figure 1(c), lanes 3 and 4 are similar to lanes 5 and 6, respectively. Although the authors provided the journal with the underlying blots for all the figures, the journal and authors decided to retract the manuscript. The replacement blot for Figure 2(b) and the underlying blots for all figures are available in the Supplementary Materials. The authors stated that the representative loading control bands in Figures 1(c) and 2(b) were improperly assembled, leading to repetitions of bands, and that these errors do not affect the results, once several papers have demonstrated that exercise does not change both, ACC [10, 11] and actin [12–14] protein content, in the skeletal muscle of rodents. In light of the figure preparation issues, the authors sincerely apologize to the scientific community for any misunderstanding that these errors may have caused.
